# *In vitro* and *In silico* Models to Study SARS-CoV-2 Infection: Integrating Experimental and Computational Tools to Mimic “COVID-19 Cardiomyocyte”

**DOI:** 10.3389/fphys.2021.624185

**Published:** 2021-02-17

**Authors:** Rafael Dariolli, Chiara Campana, Amy Gutierrez, Eric A. Sobie

**Affiliations:** Department of Pharmacological Sciences, Icahn School of Medicine at Mount Sinai, New York, NY, United States

**Keywords:** COVID-19, SARS-CoV-2, cardiomyocytes, hiPSC-CMs, modeling, pluripotent stem cells

## Abstract

The rapid dissemination of SARS-CoV-2 has made COVID-19 a tremendous social, economic, and health burden. Despite the efforts to understand the virus and treat the disease, many questions remain unanswered about COVID-19 mechanisms of infection and progression. Severe Acute Respiratory Syndrome (SARS) infection can affect several organs in the body including the heart, which can result in thromboembolism, myocardial injury, acute coronary syndromes, and arrhythmias. Numerous cardiac adverse events, from cardiomyocyte death to secondary effects caused by exaggerated immunological response against the virus, have been clinically reported. In addition to the disease itself, repurposing of treatments by using “off label” drugs can also contribute to cardiotoxicity. Over the past several decades, animal models and more recently, stem cell-derived cardiomyocytes have been proposed for studying diseases and testing treatments *in vitro*. In addition, mechanistic *in silico* models have been widely used for disease and drug studies. In these models, several characteristics such as gender, electrolyte imbalance, and comorbidities can be implemented to study pathophysiology of cardiac diseases and to predict cardiotoxicity of drug treatments. In this Mini Review, we (1) present the state of the art of *in vitro* and *in silico* cardiomyocyte modeling currently in use to study COVID-19, (2) review *in vitro* and *in silico* models that can be adopted to mimic the effects of SARS-CoV-2 infection on cardiac function, and (3) provide a perspective on how to combine some of these models to mimic “*COVID-19 cardiomyocytes environment.*”

## Introduction

Since the first reported case in Wuhan, China on December 31st, 2019, the Severe Acute Respiratory Syndrome coronavirus 2 (SARS-CoV-2) has precipitated the coronavirus disease 2019 (COVID-19) pandemic, a global socio-economic and health burden (Bialek et al., 2020; Guan et al., 2020). As of January 08th, 2021, the total number of confirmed cases reported is approximately 86 million with more than 1.8 million deaths registered (WHO, 2020). The disease can affect most of the population with factors such as age, gender, race, socioeconomic status affecting prognosis (Gebhard et al., 2020; Golestaneh et al., 2020; Sharma G. et al., 2020; Zhou F. et al., 2020). Additionally, pre-existing cardiovascular diseases (CVDs) such as hypertension, diabetes, and heart failure are prevalent in cohorts of patients with the most serious forms of COVID-19 (Goyal et al., 2020; Grasselli et al., 2020; Guan et al., 2020; Huang C. et al., 2020; Wu and McGoogan, 2020).

At the cellular level, SARS-CoV-2 infects its target by engaging with the angiotensin-converting enzyme 2 (ACE2) receptor, followed by cleavage of the viral spike (S) protein by the host serine protease TMPRSS2. Once in the cytoplasm, the viral RNA is replicated and released via exocytosis causing cellular damage (Hoffmann et al., 2020). In fact, human RNA sequencing has shown that the expression of both ACE2 and TMPRSS2 can be found in multiple organs (Chen et al., 2020), suggesting that SARS-CoV-2 might infect several target tissues.

Similar to the viruses responsible for the 2003 SARS outbreak and the 2012 MERS outbreak (Yu et al., 2006; Alhogbani, 2016), SARS-CoV-2 can trigger cardiovascular illnesses such as thromboembolism, myocardial injury, acute coronary syndromes, and arrhythmias (Clerkin et al., 2020; Madjid et al., 2020; Zheng et al., 2020). The heart is composed of many cell types including cardiomyocytes, endothelial cells, pericytes, epithelial cells, fibroblasts, smooth muscle cells, and immune cells (Pinto et al., 2016; Zhou and Pu, 2016; Chen et al., 2020) with all cell types contributing in some way to the overall cardiac function (Borg et al., 1996). Given the central role of cardiomyocytes in force generation and the minimal regenerative capacity of these cells (Cohn et al., 2000), avoiding cardiomyocyte damage and loss is of paramount importance to survival.

In the context of COVID-19, patients with poor prognosis who require hospitalization tend to exhibit a high prevalence of CVDs before viral infection. To understand the cardiovascular manifestations of COVID-19 and its treatments, experimental and mathematical cardiomyocyte models are likely to be of value. In this Mini Review, we: (1) present the state of the art of *in vitro* and *in silico* models of cardiomyocytes currently in use to study COVID-19, (2) review *in vitro* and *in silico* models that can be adopted to mimic the effects of SARS-CoV-2 infection on cardiac function, and (3) propose a perspective on how to create robust models that resemble a “*COVID-19 cardiomyocyte environment*” though the combination of *in vitro* and *in silico* strategies.

## Modeling the “COVID-19 Cardiomyocyte” *in vitro*

In some of the first autopsies of deceased COVID-19 patients, electron microscopy identified viral particles compatible with the *Coronaviridae* family in multiple cardiac cell types, including cardiomyocytes, endothelial cells, macrophages, neutrophils, and fibroblasts (Dolhnikoff et al., 2020; Fox et al., 2020a,b; Lax et al., 2020; Lindner et al., 2020). Despite this preliminary evidence, the mechanism of direct infection of human adult cardiomyocytes is still not completely elucidated.

A recent single-cell sequencing of adult hearts demonstrated that expression of ACE2 receptors is higher in pericytes than in cardiomyocytes (Chen et al., 2020). Additionally, neither pericytes nor cardiomyocytes seem to significantly express the protease TMPRSS2 (Litviňuková et al., 2020), strongly suggesting that, if SARS-CoV-2 does in fact enter cardiomyocytes, it may do so through a pathway other than ACE2/TMPRSS2 (Pérez-Bermejo et al., 2020; Yang and Shen, 2020). Further, the high expression of ACE2 receptors in endothelial cells suggests that they represent a likely source of SARS-CoV-2 infection. In fact, the infection of endothelial cells by SARS-CoV-2 results in blood vessel inflammation (endotheliitis) in multiple organs, including the heart (Varga et al., 2020).

In this context, the use of *in vitro* models has been proposed in an effort to overcome the limitations related to the use of human tissues *post-mortem* (Yang et al., 2020). Human induced pluripotent stem-cell derived cardiomyocytes (hiPSC-CMs) can be directly infected by SARS-CoV-2 (Bojkova et al., 2020; Marchiano et al., 2020; Sharma A. et al., 2020; Yang et al., 2020). These infected cells display impairment of their spontaneous beating behavior (Bojkova et al., 2020; Marchiano et al., 2020; Sharma A. et al., 2020). Additionally, an excessive increase of caspase-3 cleavage, which drives cells to an apoptotic program, has been reported in these cells (Bojkova et al., 2020; Sharma A. et al., 2020).

Recent reports have suggested that hiPSC-CMs are infected via an alternative route involving the ACE2 receptor and cathepsin-dependent endolysosomes (Bojkova et al., 2020; Marchiano et al., 2020; Pérez-Bermejo et al., 2020), rather than through TMPRSS2 protease cleavage (Hoffmann et al., 2020). In fact, hiPSC-CMs display low expression of TMPRSS2 while cathepsins-L and -B, cysteine proteases which are also able to mediate priming of the viral S-protein (Hoffmann et al., 2020), are highly expressed in these cells (Bojkova et al., 2020). Furthermore, the block of cathepsins by chemical inhibition results in significant reduction of viral particles in hiPSC-CMs (Bojkova et al., 2020; Pérez-Bermejo et al., 2020).

In addition, SARS-CoV-2 exposure induces significant transcriptional changes resulting in the disruption of the contractile apparatus of hiPSC-CMs (Pérez-Bermejo et al., 2020). These cytopathic effects progressively affect hiPSC-CM electrophysiological and contractile properties as recently demonstrated. Microelectrode array measurements of hiPSC-CMs infected with SARS-CoV-2 documented a significant increase in their field potential duration (Marchiano et al., 2020), an *in vitro* surrogate for arrhythmogenicity. Similarly, infected three-dimensional engineered heart tissues displayed progressive impairment of contractility suggesting a disruption of the contractile apparatus following infection with SARS-CoV-2, which may contribute to whole-organ dysfunction (Huang L. et al., 2020; Marchiano et al., 2020).

Despite the exciting results describing the direct infection of cardiomyocytes by SARS-CoV-2, increasing clinical evidence points toward the indirect effects of the infection accounting for the most prevalent and severe cases that exhibit cardiac repercussions (Clerkin et al., 2020; Zheng et al., 2020). The field currently lacks robust models that can clarify these aspects of COVID-19 at the cardiomyocyte level.

Rising evidence shows that COVID-19 patients with worse prognosis present cardiac damage that correlates with the concentration of pro-inflammatory molecules (Akhmerov and Marbán, 2020; Ruan et al., 2020; Zhou F. et al., 2020). Indeed, severe symptoms, mainly related to the hyperinflammation and deficit of oxygenation, have been described in the most aggressive cases (Iannaccone et al., 2020; Lax et al., 2020). The inability to promptly defeat a viral infection can elicit a *cytokine storm*, in which pro-inflammatory molecules including Interleukin-1β (IL-1β), Interleukin-6 (IL-6), and Tumor necrosis factor (TNF-α) are released in pathogenic concentrations causing systemic hyperinflammation (Iannaccone et al., 2020).

Pro-inflammatory molecules can directly cause adverse consequences in cardiomyocytes including arrhythmias (Long, 2001; El Khoury et al., 2014; Aromolaran et al., 2018; Keck et al., 2019), cellular hypertrophy (Long, 2001; Carreño et al., 2006; Smeets et al., 2008), and cell death (Wang et al., 2016). Elevated levels of IL-1β can trigger cardiac arrhythmias through the impairment of expression of proteins that control calcium handling, ultimately affecting cardiomyocyte’s contraction (McTiernan et al., 1997; El Khoury et al., 2014). Similarly, neonatal cardiomyocytes (NCs) exposed to IL-6 in culture display augmentation of cell size suggesting cellular hypertrophy (Hirota et al., 1995). Interestingly, NCs co-cultured with fibroblasts overexpressing IL-6 are driven to apoptosis (Wang et al., 2016). Furthermore, IL-6 converts cardiac fibroblasts into myofibroblasts which produce collagen contributing to the formation of fibrosis (Wang et al., 2016). Pathological levels of IL-6 cause down-regulation of hERG channel (ether-a-go-go-related gene) expression, resulting in increased risk of action potential duration (APD) prolongation and arrhythmias (Aromolaran et al., 2018).

TNF-α is another pro-inflammatory molecule that triggers cardiac arrythmias and induces cardiomyocytes’ hypertrophy and death (Nakamura et al., 1998; Carreño et al., 2006; Shen et al., 2018). Rat NCs treated with TNF-α exhibit abnormal size (Nakamura et al., 1998). Further, pathological levels of TNF-α can enhance mitochondrial fragmentation, promoting cell death (Shen et al., 2018), and a slow and sustained increase in hypertrophic markers through the NF-Kβ pathway (Smeets et al., 2008).

The *cytokine storm* caused by SARS-CoV-2 triggers an acute respiratory distress syndrome (ARDS) resulting in severe outcomes such as oxygen deprivation (hypoxia) and electrolyte disturbance (e.g., hypokalemia), factors that cause cardiomyocyte distress (Bhatia et al., 2012; Coperchini et al., 2020; Xu et al., 2020). Moreover, it appears that this hypoxia may induce release of additional cytokines, potentially leading to further myocyte dysfunction. In isolated rat NCs subjected to hypoxia (5% O_2_), production and release of IL-6 are enhanced (Yamauchi-Takihara et al., 1995; Wang et al., 2016). In addition, conditioned media from rat NCs cultivated at 1% O_2_ exhibit higher levels of TNF-α, IL-1β, IL-6, and transforming growth factor beta (TGF-β) compared to cells cultivated in normoxia (Shi et al., 2017).

Electrolyte imbalance and fever are two other typical conditions implicated in COVID-19 patients. Several models of hypokalemia and hyperthermia have indicated that slight changes in the cellular environment can dramatically impair cardiomyocytes’ stability (El-Battrawy et al., 2016; Weiss et al., 2017; Tazmini et al., 2020). Hypokalemia is a systemic decrease in the concentration of K^+^ ions that can produce APD prolongation and arrhythmias in cardiomyocytes (Weiss et al., 2017), including hiPSC-CMs (Kuusela et al., 2017). In addition, arrhythmias associated with hyperthermia have also been reported in both healthy and ill individuals (Saura et al., 2002; Pasquié et al., 2004; Burrell et al., 2007), and cellular studies have reported reductions in important cardiac ion channels caused by hyperthermia (El-Battrawy et al., 2016).

Taken together, the previous reports provide substantial information on how to model several outcomes that account for the cardiac deterioration observed in many COVID-19 patients. Studies that use patient-derived hiPSC-CMs carrying inherited diseases can also be found in the literature (Granéli et al., 2019; Hoes et al., 2019; Jimenez-Tellez and Greenway, 2019). Several of these models can be adopted to evaluate additive effects of COVID-19 and pre-existing comorbidities such as heart failure, cardiomyopathies, diabetes. The most representative *in vitro* models that mimic COVID-19 outcomes, as well as some of the significant hiPSC lines derived from patients with pre-existing comorbidities are described in Table 1.

**TABLE 1:** *In vitro* models to be used to mimic “COVID-19 cardiomyocytes.”

Outcome	Stimuli	Treatment	Specie	Cell type	References
Inflammation/Hypertrophy	IL-1β	1 ng/mL, 12–16 h (adult cells) and 30 pg/mL 24–32 h	mouse	neonatal ventricular myocytes and adult ventricular myocytes	El Khoury et al., 2014
	TNF-α	1 ng/mL, 12–16 h and 30 pg/mL 24–32 h			
	TNF-α	50 ng/mL, 2, 12, 24, and 48 h	rat	Neonatal cardiomyocytes	Smeets et al., 2008
	TNF-α	1, 10, and 100 ng/mL (centered in 10 ng/mL), from 1 hour to 3 days.	rat	Neonatal cardiomyocytes	Nakamura et al., 1998
	TNF-α	5, 10, and 20 ng/mL, 6 or 48 h	rat	H9C2 rat cardiomyocytes	Shen et al., 2018
	IL-6	5, 10, and 20 ng/mL, 6 or 48 h			
	IL-6	2 μg/mL for 72 h	mouse	neonatal cardiomyocytes	Hirota et al., 1995
	IL-6	20 ng/mL for 40 min	guinea-pig	adult ventricular myocytes	Aromolaran et al., 2018
Hypoxia	Gases concentration	95% N_2_ – 5% CO_2_ (different regimens of time)	rat	neonatal ventricular cardiomyocytes	Yamauchi-Takihara et al., 1995
	Gases concentration	not described	rat	neonatal ventricular cardiomyocytes	Wang et al., 2016
	Gases concentration and culture medium	nitrogen equilibrated DMEM and 1% O_2_ and 5% CO_2_ for 2, 4, 6, 8, 10, and 12 h	mouse	neonatal cardiomyocytes	Shi et al., 2017
	Gases concentration	1% O_2_ (adjusted by N_2_ replacement in different regimens of time)	human/chimpanzee	iPSC-CMs	Ward and Gilad, 2019
	Gases concentration	1% O_2_ (adjusted by N_2_ replacement)	human	iPSC-CMs	Plant et al., 2020
Electrolyte imbalance (hypokalemia)	K^+^ concentration in the medium	5.33, 4, 3, 2 and 1 mM of K^+^ (adjusted by adding KCl into a K^+^ free medium)	human	iPSC-CMs	Kuusela et al., 2017
	K^+^ concentration in buffer solution	from 5 to 2.7 mmol/L rapidly reduction of K^+^ superfusion	rat	atrial and ventricular adult myocytes	Tazmini et al., 2020
Hyperthermia	Temperature increase	36 vs. 40°C	human	iPSC-CMs	El-Battrawy et al., 2016
HCM	MYH7	missense mutation (Arginine442Glycine)	human	iPSC-CMs	Han et al., 2014
	MYBPC3	c.2373dupG mutation	human	iPSC-CMs	Birket et al., 2015
	MYBPC3	heterozygous c.1358-1359insC	human	iPSC-CMs	Prondzynski et al., 2017
DCM	phospholamban (PLN)	R14del mutation	human	iPSC-CMs	Stillitano et al., 2016
	LMNA gene	R225X, Q354X, and T518fs patient mutation	human	iPSC-CMs	Lee et al., 2017

## Modeling the “COVID-19 Cardiomyocyte” *in silico*

Besides the use of *in vitro* strategies, many authors have been reporting *in silico* models to study COVID-19. Most of them are concerned with predictions of mortality and risk factors (Scheiner et al., 2020; Wicik et al., 2020; Yadaw et al., 2020), disease infection and spread (Ivorra et al., 2020; Zeb et al., 2020), and drug-target interactions (Ciliberto and Cardone, 2020; Iqbal Choudhary and Shaikh, 2020; Muthuramalingam et al., 2020; Zhou Y. et al., 2020). Regarding the cardiac repercussions of COVID-19 and its potential treatments, mechanistic approaches based on dynamical models have been proposed to predict effectiveness of treatments (Iqbal Choudhary and Shaikh, 2020; Peterson, 2020), and to measure the toxicity of repurposed drugs (Sutanto and Heijman, 2020; Varshneya et al., 2020).

Notably, several drugs currently under testing have been previously reported to cause toxicity (Chary et al., 2020; Saleh et al., 2020; Smith et al., 2020; Zhang et al., 2020). Particular attention needs to be paid to drugs that can substantially increase arrhythmia risk or increase the risk of heart failure (Michaud et al., 2020), as addressed in a few recent studies. For example, *Sutanto and Heijman* used a canine ventricular cardiomyocyte model to simulate action potentials (APs) of cardiomyocytes treated with chloroquine (CQ) and azithromycin (AZM). The authors demonstrated that β-adrenergic stimulation is protective against CQ- and AZM-induced proarrhythmia by preventing APD prolongation and afterdepolarizations (Sutanto and Heijman, 2020). Meanwhile, our group has combined pharmacokinetics (PK) and electrophysiology modeling of human ventricular cardiomyocytes to predict the risk of potential cardiac adverse events caused by CQ, AZM, lopinavir (LP), and ritonavir (RT). Our simulations showed treatment with clinically relevant doses of CQ/AZM was more dangerous than treatment with LP/RT, and that females with pre-existing heart failure were at the highest risk of developing ventricular arrhythmia from drug treatments (Varshneya et al., 2020). These studies (Sutanto and Heijman, 2020; Varshneya et al., 2020) suggests that future work needs to address how pre-existing diseases and COVID-19 clinical presentation (e.g., hyperinflammation, fever, ion imbalance) may affect arrhythmia susceptibility.

The use of *in silico* models to simulate electrophysiological perturbations and to predict disease severity and treatment efficacy is a mature field of research (Lancaster and Sobie, 2016; Passini et al., 2017; Jæger et al., 2019a,b; Li et al., 2019; Gando et al., 2020). Dynamic models of cardiomyocyte’s electrophysiology are particularly useful for simulating between-patient variability (Muszkiewicz et al., 2016), allowing the study of phenotypic minorities, such as high-risk COVID-19 patients (Varshneya et al., 2020). This variability among individuals is hard to replicate in other model types (e.g., *in vivo*, *in vitro*). Further, *in silico* approaches can provide a quick illustration of how multiple factors in combinations (e.g., *cytokine storm* plus pre-existing diseases plus drug-treatments), can exacerbate positive or negative outcomes.

Over the past decades numerous *in silico* models that resemble electrophysiological properties of cardiomyocytes have been proposed based on experimental data from different species (Krogh-Madsen et al., 2016; Mayourian et al., 2018). These models can be used to highlight physio- and pathophysiological characteristics of ion channels and cellular compartments, as well as their intricate relationships, in order to gain a mechanistic understanding of a variety of illnesses and drug-treatments (Pandit et al., 2003; Sarkar and Sobie, 2011; Petkova-Kirova et al., 2012; Atkinson et al., 2016; Devenyi and Sobie, 2016; Das et al., 2017; Paci et al., 2018; Varshneya et al., 2018; Gong et al., 2020; Sutanto et al., 2020). However, only few models that mimic inflammation (Petkova-Kirova et al., 2012) and hyperthermia (Atkinson et al., 2016) can be found in the literature, posing barriers to the modeling of COVID-19 secondary effects on cardiomyocytes. Further, many of these models are based on animal experiments, limiting translation of their findings.

It is worth mentioning that most of the models previously cited are based on ordinary differential equations (ODEs) and describe the action potential and calcium transient of an isolated cardiomyocyte. Therefore, these models cannot assess how interactions among cardiomyocytes as well as with fibroblasts and other cell types contribute to the overall cardiac function. These shortcomings can be addressed by bi-(2D) and tridimensional (3D) models (e.g., fiber, tissue), where electrical coupling and the resultant tissue-level behavior can be simulated (Lines et al., 2002; Jæger et al., 2019a; Hwang et al., 2020). These models can be used to investigate mechanisms of cardiac arrhythmia, such as cardiac reentry that can be induced by Early After Depolarizations (EADs). Complex models are promising and can be particularly beneficial considering correlations with experimental data obtained from 3D hiPSC-CM models (e.g., field potential assessments by MEA in hiPSC-CM monolayers or 3D structures) (Kügler, 2020). However, these spatial models are more computationally expensive and are less frequently used than single-cell models (Jæger et al., 2019a). The most significant *in silico* models able to partially simulate COVID-19 effect in cardiomyocytes are listed in Table 2.

**TABLE 2:** *In silico* models to be used to mimic “COVID-19 cardiomyocytes.”

Outcome	Stimuli	Treatment/Simulation	Specie	Cell type	References
Drug-treatment (β-adrenergic signaling)	healthy cells under sympathetic stimulation	CQ, AZM	dog	ventricular cardiomyocytes	Sutanto and Heijman, 2020
Drug-treatment, heart failure, gender	healthy cells and heart failure cells from male and female	CQ, AZM, LP, RT	human	endocardial ventricular myocytes	Varshneya et al., 2020
Drug-treatment	healthy cells	Several drugs	human	ventricular cardiomyocytes	Lancaster and Sobie, 2016
Drug-treatment	healthy cells	several drugs	human	ventricular cardiomyocytes	Passini et al., 2017
Drug-treatment	healthy cells/dynamic hERG submodels	several drugs	human	ventricular cardiomyocytes	Li et al., 2019
Genetic disease (Q1475P Na_v_ 1.5 mutation)	healthy cells modified by Markov model for fast and late Na^+^ current	Na_v_1.5 mutation	human	endocardial ventricular myocytes	Gando et al., 2020
Comorbidity (diabetes type-I)	streptozotocin-induced, type-I diabetes in rats	baseline model vs. diabetes model	rat	right ventricle cardiomyocytes	Pandit et al., 2003
Ion current changes	75% block of I_Kr_	baseline vs. I_Kr_ blocked cells	dog and human	ventricular cardiomyocytes	Sarkar and Sobie, 2011
Arrhythmogenic susceptibility	changes in the conductances of I_Kr_ and I_Ks_	baseline vs. I_Kr_ and I_Ks_ modified cells	several	ventricular cardiomyocytes	Varshneya et al., 2018
β-adrenergic signaling/activity	healthy cells under sympathetic	human model parametrization for β-adrenergic system	human	epicardial ventricular cardiomyocytes	Gong et al., 2020
Inflammation/Hypertrophy	TNF-α overexpression in the heart	Parameterization using cardiomyocytes isolated from hearts overexpressing TNF-α	mouse	apical ventricular cardiomyocytes	Petkova-Kirova et al., 2012
Hyperthermia (fever)	Fever	baseline vs. adjusted model for fever based on malaria	human	atrial and ventricular cardiomyocytes	Atkinson et al., 2016
Drug-treatments, model validation, ion current changes	healthy cells vs. modifications: physiological and cardiotoxic spectrum	Tetrodotoxin, nifedipine, 3R4S-Chromanol 293B, E4031	human	Atrial and ventricular hiPSC-CMs	Paci et al., 2013
Ion imbalances, heart failure, hiPSC-CM/adult cardiomyocytes predictions	healthy cells vs. a variety of conditions and species cross predictions and validations	ion channel blocks, ion buffer composition changes, pacing rates, heart failure	human, guinea pig, rabbit	iPSC-CMs (human) and adult cardiomyocytes (different species)	Gong and Sobie, 2018
hiPSC-CM/adult cardiac microtissues, Drug-treatments	healthy microtissues from hiPSC-CMs	Cisapride and verapamil treatments (different doses)	human	iPSC-CMs (human)/adult myocytes	Tveito et al., 2018
hiPSC-CM/adult cardiac microtissues, Drug-treatments	healthy microtissues from hiPSC-CMs	Cisapride, verapamil, lidocaine, nifedipine, flecainide (many dose)	human	iPSC-CMs (human)/adult myocytes	Jæger et al., 2020

## Discussion/Perspective

Despite the rapid dissemination of high-quality science during the COVID-19 pandemic, crucial gaps of knowledge remain open. Here, we (1) reviewed the most up to date protocols used to study COVID-19 effects in cardiomyocytes, and (2) reviewed several *in vitro* and *in silico* models of inflammation, ischemia/hypoxia, hyperthermia, hypokalemia, and hypertrophy, with great relevance cardiovascular effects of COVID-19 that are not due to direct infection of cardiomyocytes by SARS-CoV-2.

In this scenario, hiPSC-CMs emerge as a promising platform for modeling COVID-19. However, these cells display limited maturation and biological heterogeneity, partly due to a lack of consensus protocols for their generation and characterization, negatively contributing to their clinical translation (Lundy et al., 2013; Robertson et al., 2013; Koivumäki et al., 2018; Gintant et al., 2019; Hoang et al., 2019; Ribeiro et al., 2019). Several strategies have been proposed to overcome hiPSC-CMs maturation obstacles. Nonetheless, most of them only result in limited improvement, especially in the case of 2D models (Talkhabi et al., 2016; Sun and Nunes, 2017). In parallel, 3D models such as cardiac spheroids (Polonchuk et al., 2017; Mattapally et al., 2018) and “*engineered heart tissues*” (EHTs) (Nunes et al., 2013; Stoehr et al., 2014) have shown promising results toward obtaining mature cells and a phenotype that more closely resembles adult tissue. However, the specialized expertise required for 3D technologies and the expense of these assays remains a challenge that limits the use of these approaches for research groups that require scalable or high throughput implementation (Zuppinger, 2019).

Additionally, *in silico* models of hiPSC-CMs’ electrophysiology became a reality (Paci et al., 2013, 2018; Gong and Sobie, 2018; Tveito et al., 2018; Jæger et al., 2020), allowing the simulation of disease effects and drug toxicity (Gong and Sobie, 2018; Jæger et al., 2020). Similarly to the case of experimental models, there are peculiarities and limitations for modeling cardiomyocytes *in silico* (Gong et al., 2017). However, *in silico* models have the flexibility of being easily adapted to new experimental data, such as the ones obtained from hiPSC-CMs, allowing for more accurate quantitative predictions (Lei et al., 2017; Jæger et al., 2020; Paci et al., 2020). Furthermore, the most recent mechanistic models permit an improved translation of electrophysiological findings from hiPSC-CMs to human adult myocytes at both single-cell and tissue level (Gong and Sobie, 2018; Tveito et al., 2018). Overall, these strategies consider proportional changes in proteins expression throughout maturation without significant changes in the cell’s function. Therefore, regression models, among other strategies can be used to parameterize ion current densities and correlate hiPSC-CM to adult cardiomyocyte models (Gong and Sobie, 2018; Tveito et al., 2018).

Currently, universal protocols for the generation and characterization of hiPSC-CMs are not available. Depending on their application, strengths and weaknesses exist for both 2D and 3D models (Zuppinger, 2019). Especially in the context of a pandemic, strengths, and weaknesses should be pondered to allow for fast and meaningful experimental research. The best models of choice in this scenario are the ones that can generate accurate results but in a timely fashion. Independently of the model of choice, experiments need to be conducted in well-controlled environment, replicated for different cell lines, and always accompanied by negative controls (non-treated, healthy). Analogously, *in silico* models should be chosen to best match the experimental approach. The interpretation of results needs to be cautious, always considering the intrinsic limitations of each model.

Thus, the integration of experimental data obtained from hiPSC-CMs (single-cell, 2D, and 3D models) with appropriate *in silico* models that can quantitatively predict functional cardiac outcomes in adult cells is of paramount importance. hiPSC-CMs from healthy donors or patients with pre-established comorbidities can be utilized to investigate *in vitro* the reaction of cardiomyocytes to several conditions precipitated by the systemic effects of SARS-CoV-2 infection. Many of these models were discussed in this mini review. The results obtained from experiments with hiPSC-CMs will provide valuable information that can be integrated into *in silico* models and used to predict disease progression and the effects of treatment.

In conclusion, we presented a perspective on how to combine *in vitro* and *in silico* approaches to generate human-based platforms to study COVID-19 repercussions on the cardiomyocyte’s function. The use of robust and precise models and their integration in mechanistic platforms may contribute substantially to understanding the impact of COVID-19 and COVID-19 drug treatments on the heart, constituting an additional source of guidance to help clinicians in the front line.

## Author Contributions

RD conceived the manuscript and wrote the initial draft. CC, AG, and ES helped to edit the manuscript. All authors contributed to the article and approved the submitted version.

## Conflict of Interest

The authors declare that the research was conducted in the absence of any commercial or financial relationships that could be construed as a potential conflict of interest.
